# Assessing the Risk of Postoperative Delirium Through Comprehensive Geriatric Assessment and Eastern Cooperative Oncology Group Performance Status of Elderly Patients With Gastric Cancer

**DOI:** 10.1245/s10434-024-16034-w

**Published:** 2024-10-07

**Authors:** Takefumi Itami, Kazuyoshi Yamamoto, Yukinori Kurokawa, Takuro Saito, Tsuyoshi Takahashi, Kota Momose, Kotaro Yamashita, Koji Tanaka, Tomoki Makino, Yukiko Yasunobe, Hiroshi Akasaka, Taku Fujimoto, Koichi Yamamoto, Kiyokazu Nakajima, Hidetoshi Eguchi, Yuichiro Doki

**Affiliations:** 1 Department of Gastroenterological Surgery, Osaka University Graduate School of Medicine, Suita, Osaka, Japan; 2 Department of Geriatric and General Medicine, The University of Osaka Graduate School of Medicine, Osaka, Japan; 3 Department of Rehabilitation Science, Osaka Health Science University, Osaka, Japan; 4 Department of Hygiene and Preventive Medicine, Iwate Medical University, Idaidori, Yahaba, Iwate Japan

**Keywords:** Gastric cancer, Gastrectomy, Elderly patients, Comprehensive geriatric assessment, Delirium, Mini-Mental State Examination, Eastern Cooperative Oncology Group performance status

## Abstract

**Background:**

Postoperative delirium is especially common and often problematic among elderly patients undergoing surgery. This study aimed to explore factors that can predict postoperative delirium in elderly patients undergoing gastric cancer surgery.

**Methods:**

This cohort study included 255 patients age 75 years or older who underwent gastric cancer surgery between July 2010 and December 2020. All the patients underwent preoperative comprehensive geriatric assessment (CGA) evaluation by a geriatrician. In addition to the CGA items, this study investigated the association between postoperative delirium and clinicopathologic factors, including Eastern Cooperative Oncology Group performance status (ECOG-PS).

**Results:**

The most common postoperative complication was delirium, present in 31 patients (12.2%). The group with delirium was significantly more likely to have ECOG-PS ≥ 2, diabetes mellitus, cardiovascular disease, or cerebral infarction. The CGA showed frailty in the Instrumental Activities of Daily Living scale (IADL), the Mini-Mental State Examination (MMSE), the Vitality Index (VI), and the Geriatric Depression Scale 15 (GDS-15). In the multivariate analysis, the independent risk factors for delirium were ECOG-PS ≥ 2 (*P *= 0.002) and MMSE-frailty (*P *< 0.001). Using an MMSE score of ≤ 23 and an ECOG-PS score of ≥ 2 as cutoffs, postoperative delirium was predicted with a sensitivity of 80.7% and a specificity of 74.1%.

**Conclusion:**

Postoperative delirium might be more easily predicted based on the combination of MMSE and ECOG-PS for elderly patients with gastric cancer undergoing gastrectomy.

As the number of elderly individuals in the population increases, opportunities for elderly individuals to undergo surgery continue to increase. Postoperative complications are known to affect life expectancy, decrease quality of life, and prolong hospital stays.^1–3^ Elderly patients have been reported to experience postoperative complications even more frequently than typical-age patients.^4^ Therefore, it is important to assess perioperative risks correctly in elderly patients.

However, with the increase in the number of elderly people, the degree of comorbidity and frailty among the elderly varies greatly from person to person, making it difficult to determine true frailty based on age alone. Thus, it is important to assess frailty in the elderly from multiple perspectives using the comprehensive geriatric assessment (CGA).^5^ The CGA has gradually gained worldwide acceptance since Rubenstein et al.^6^ first described it in 1984 as a tool to improve life and functional prognosis in the elderly.

The CGA is a multifaceted tool for assessing physical, psychological, and social aspects and comorbidities in elderly patients. Various studies have evaluated the impact of CGA on postoperative complications and mortality.^7,8^

Postoperative delirium is a known complication that often occurs in the elderly. The incidence of delirium after major non-cardiovascular surgery ranges from 13 to 50%.^9^ Postoperative delirium also is associated with longer hospital stays, morbidity, mortality, and higher health care costs.^10^ Furthermore, it has been reported that surgical patients with delirium experience cognitive decline. This impairment can persist up to 1 year after surgery.^11^ Because advances in gastric cancer treatment might lead to more elderly patients receiving postoperative chemotherapy or immunotherapy in the future,^12,13^ delayed postoperative recovery due to delirium might have a negative impact on treatment. Therefore, it is crucial to assess the risk of postoperative delirium accurately in elderly patients.

We previously reported on the association between postoperative delirium and CGA in elderly patients with esophageal or colorectal cancer,^14,15^ but to date, no studies have investigated elderly patients undergoing gastrectomy for gastric cancer. In addition, because the CGA has many items and requires evaluation by a geriatrician, it is time-consuming and cumbersome. Therefore, development of a simpler and more effective method of predicting delirium is needed.

We have been performing routine preoperative CGA assessments by geriatricians for elderly patients with gastric cancer since July 2010. This study aimed to ascertain the most convenient and predictive factors of delirium, including individual components of the CGA and Eastern Cooperative Oncology Group performance status (ECOG-PS).

## Methods

### Patients

This cohort study included consecutive patients age 75 years or older who underwent gastrectomy for gastric cancer at Osaka University Hospital between July 2010 and December 2020. The study excluded patients who did not undergo CGA evaluation, failed to achieve R0 resection, or underwent local gastrectomy, subtotal esophagectomy, or concurrent surgery for another cancer. This study was approved by the institutional review board of Osaka University Hospital (certificate no. 13350T1-6).

### Preoperative CGA

The CGA was routinely performed for each patient before hospitalization or during the pre-surgical hospitalization period by a geriatrician from the Department of Geriatrics at Osaka University Hospital. The choice of assessment tools was based on several recent studies on the evaluation of geriatric patients undergoing surgery.^14–16^ In this study, the CGA consisted of five assessments: the Barthel Index and Instrumental Activities of Daily Living scale (IADL) was used to assess physical function. The Mini-Mental State Examination (MMSE), the Geriatric Depression Scale 15 (GDS-15), and the Vitality Index (VI) were used to assess mental function.

The Barthel Index assesses activities of daily living (ADLs). It consists of 10 items (eating, transferring from a sitting position to bed, grooming, toileting, bathing, walking in the hallway, climbing stairs, dressing, fecal continence, and urinary continence) rated on a scale of 5 points (range, 0–100), with higher scores indicating robustness.^17^ The IADL items are assessed at a higher level of function than ADLs and include five items for men (use of the telephone, shopping, transportation by car or public transportation, medication, and money management) and eight items for women (in addition to the five items for men, meal preparation, laundry, and ability to perform household tasks). Higher scores indicate higher function (range: 0–5 for men and 0–8 for women).^18^ The MMSE assesses cognitive function, with higher values indicating better function (range, 0–30).^19^ The GDS-15 assesses depressive status, with lower values indicating better mood (range, 0–15).^20^ The VI assesses motivation (getting up, communicating, eating, toileting, and doing rehabilitation and other activities), with higher values indicating greater motivation (range, 0–10).

In this study, the term “frailty” was used for each CGA item below the cutoff. The GDS-15 cutoff for identifying frailty is ≥ 6 of 15. Any score other than perfect on the VI, IADL, or Barthel Index was considered indicative of frailty. The MMSE cutoff for frailty was set at ≤ 23 based on previous report.^8^

### Eastern Cooperative Oncology Group Performance Status (ECOG-PS)

The ECOG-PS parameters were 0 (ability to perform activities without any difficulty, 1 (limitations in performing physically strenuous activities but ability to walk and engage in light work), 2 (ability to walk and perform all tasks within one's surroundings but inability to perform hard work, spending more than 50% of the day out of bed), 3 (ability to perform limited tasks within one's surroundings, spending more than 50% of the day in bed or a chair, 4 (complete inability to move, spending most of the day entirely in bed or a chair).^21^

### Surgical Treatment

Gastrectomy type and extent of lymph node dissection were determined according to the Japanese Gastric Cancer Treatment Guidelines.^22^ Tumor staging followed the 15th edition of the Japanese Classification of Gastric Carcinoma.

### Diagnosis of Postoperative Delirium and Other Complications

Delirium, characterized by altered consciousness with acute onset, inattention, and disorganized thinking,^23^ was diagnosed using the Confusion Assessment Method (CAM) algorithm. Two physicians from the Department of Geriatric Medicine with training in the CAM algorithm independently evaluated patients for delirium. The diagnosis of delirium was confirmed when patients met the delirium criteria in at least one CAM assessment, followed by consensus between the two physicians. Data were gathered from the day after surgery until the day before hospital discharge. Other postoperative complications were evaluated according to the Clavien–Dindo (C–D) classification.^24^

### Statistical Analysis

Associations between postoperative delirium and clinicopathologic factors were analyzed using the chi-square test for categorical variables and the Mann–Whitney *U* test for continuous variables. Variables that demonstrated statistical significance in the univariate analysis were included in a multivariate model to assess the risk of postoperative delirium. Multiple logistic regression was performed to develop models for predicting the probability of postoperative delirium. Odds ratios (ORs) for postoperative delirium were determined through multivariate analysis. Variables with a *P* lower than 0.1 in a univariate analysis were included in the multivariate analysis. Both ORs and corresponding 95% confidence intervals (CI) were calculated to show the effect of factors on delirium. Two-sided *P* values were calculated, and *P* values lower than 0.05 was considered statistically significant. All analyses were performed with SPSS software, version 24.0 (IBM Corp., Armonk, NY, USA).

## Results

### Patient Characteristics and Postoperative Complications

**Fig. 1:**
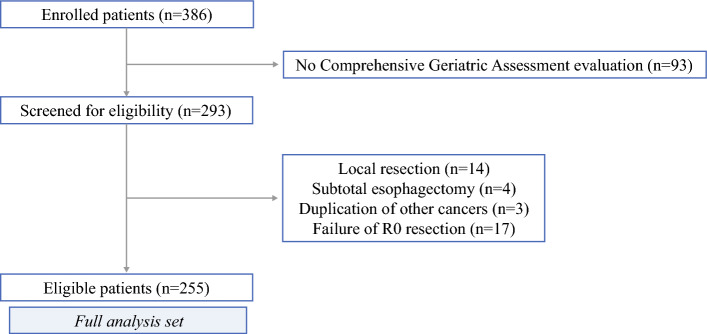
Flow chart of patient eligibility for study inclusion

During the study period, 386 patients age 75 years or older underwent gastric cancer resection. Due to scheduling issues, 93 patients were unable to undergo CGA evaluation by a geriatrician. Ultimately, 255 patients met the eligibility criteria (Fig. 1).

Patients’ background characteristics and short-term surgical outcomes are shown in Table 1. The median age was 79 years, and 72.9% of the patients were male. The study included 231 patients (90.6%) with ECOG-PS 0–1 and 24 patients (9.4%) with ECOG-PS 2–3. The most common comorbidity was hypertension (39.6%), followed by diabetes mellitus (21.2%). Among the study patients, 61.6% had cStage I disease and 1.2% had cStage IV disease.

**Table 1:** Background characteristics of the study patients

Characteristic	(*n* = 255) *n* (%)
Age (years)	
Median	79
Range	75–90
Sex	
Male	186 (72.9)
Female	69 (27.1)
BMI (kg/m^2^)	
Median	22.0
Range	14.3–30.5
ECOG-PS	
0	144 (56.5)
1	87 (34.1)
2	20 (7.8)
3	4 (1.6)
4	0 (0)
Comorbidity	
Hypertension	101 (39.6)
Diabetes mellitus	54 (21.2)
Hyperlipidemia	48 (18.8)
Coronary heart disease	47 (18.4)
Cerebrovascular disorder	37 (14.5)
Respiratory dysfunction	27 (10.6)
Neoadjuvant chemotherapy	15 (5.9)
Clinical stage	
I	157 (61.6)
II	47 (18.4)
III	48 (18.8)
IV	3 (1.2)
ASA-PS	
I	40 (15.7)
II	175 (68.6)
III	40 (15.7)
Surgical procedure type	
Open	45 (17.6)
Laparoscopic	180 (70.6)
Robot-assisted	30 (11.8)
Type of gastrectomy	
Total	45 (17.6)
Proximal	41 (16.1)
Distal	169 (66.3)
Operative time (min)	
Median	268
Range	116–632
Intraoperative blood loss (ml)	
Median	50
Range	0–1950
Postoperative level of care	
General hospital room	169 (83.6)
Intensive care unit	41 (16.1)
Postoperative complication grade ≥ II	
Delirium	31 (12.2)
Pancreatic fistula	14 (5.5)
Intra-abdominal abscess	11 (4.3)
Pneumonia	10 (3.9)
Anastomotic leak	8 (3.1)
Postoperative hospital stay (days)	
Median	16
Range	4–120
Discharge status	
Home	229 (89.8)
Rehabilitation hospital	23 (9.0)
Death	3 (1.2)

BMI, body mass index; ECOG-PS, Eastern Cooperative Oncology Group performance status; ASA-PS, American Society of Anesthesiologists physical status

Neoadjuvant chemotherapy was administered to 5.9% of the patients.

Open surgery was performed for 17.6% of the patients. All the others underwent laparoscopic or robot-assisted surgery. The most common type of gastrectomy was distal gastrectomy (66.3%), followed by total gastrectomy (17.6%). After surgery, 41 patients (16.1%) were admitted to the intensive care unit (ICU). Postoperative complications of C-D grade 2 or higher included pancreatic fistula in 14 patients (5.5%), followed by intra-abdominal abscess in 11 patients (4.3%). The median postoperative hospital stay was 16 days (range, 4–120 days), with 23 patients (9.0%) transferred to a rehabilitation hospital. Three patients (1.2%) died during postoperative hospitalization.

### CGA Components

**Fig. 2:**
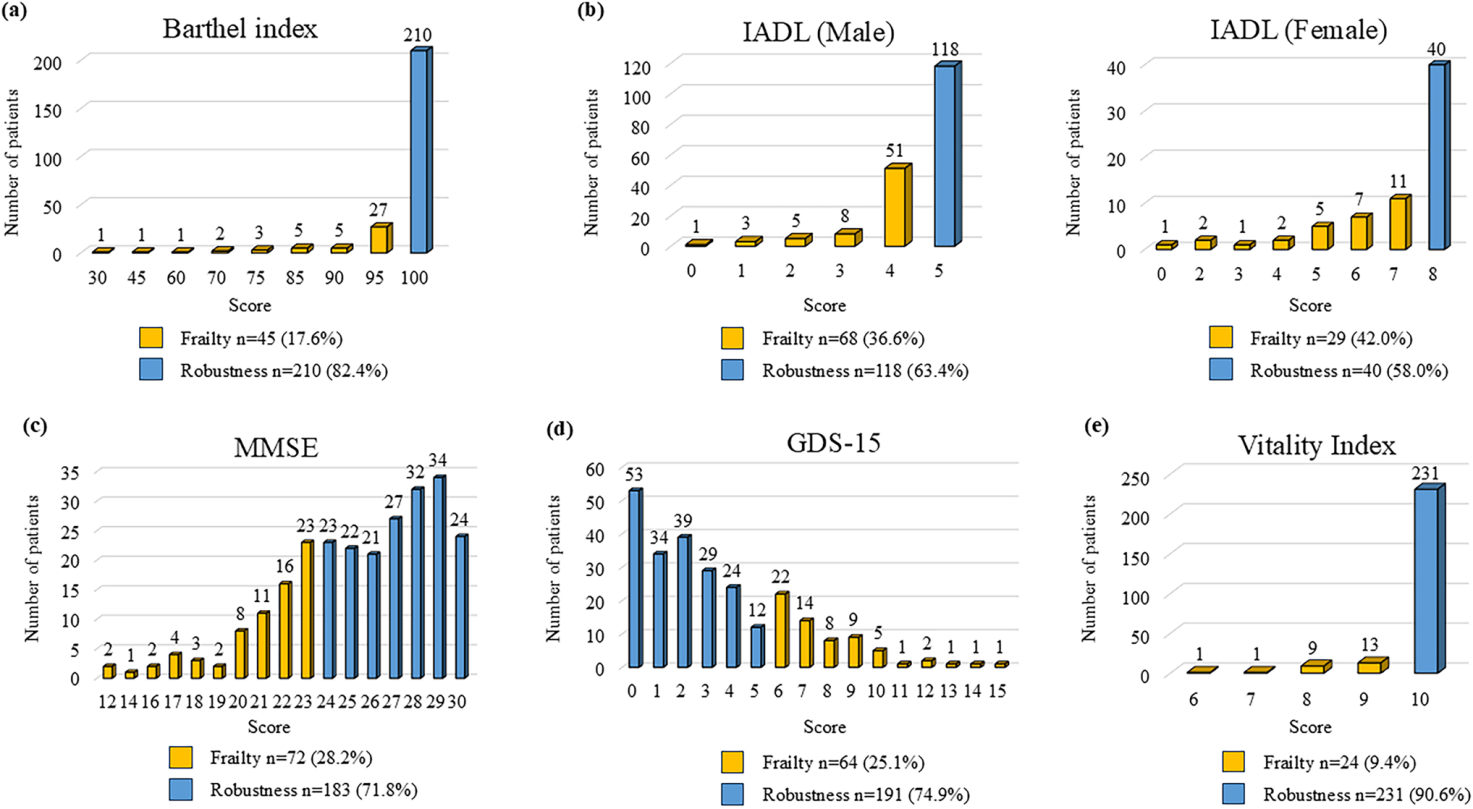
Distribution of robustness and frailty scores for each comprehensive geriatric assessment (CGA) item. **a** The Barthel Index is rated on a scale of 5 points (range, 0–100), with higher scores indicating robustness. **b** The Instrumental Activities of Daily Living scale (IADL) includes 5 items for men and 8 items for women (range: 0–5 for men, 0–8 for women), with higher scores indicating higher function. **c** The Mini-Mental State Examination (MMSE) is scored numerically (range, 0–30), with higher values indicating better function. **d** The Geriatric Depression Scale 15 (GDS-15) is scored numerically (range, 0–15), with lower values indicating better mood. **e** The Vitality Index (VI) is scored numerically (range, 0–10), with higher values indicating greater motivation. The respective cutoffs for identifying frailty were ≤23 of 30 on the MMSE, ≥6 of 15 on the GDS-15, and any score other than perfect on the VI, IADL, and Barthel Index

The interview and collection of information on CGA components required approximately 30 min. The distribution of scores for robustness and frailty for each CGA item is shown in Fig. 2. A perfect score on the Barthel Index indicated robustness for 210 patients (82.4%), and frailty was observed for 45 patients (17.6%). Regarding IADL, frailty was observed in 68 (36.6%) of the male patients and 29 (42.0%) of the female patients. The median MMSE score was 26 (range, 12–30), and 72 patients (28.2%) had a frailty score of 23 or lower. The median GDS-15 score was 3 (range, 0–15), and 64 patients (25.1%) had a frailty score of 6 or higher. Regarding VI, 231 patients (90.6%) had a perfect score.

### Adverse Events Due to Delirium

Postoperative delirium was observed in 31 patients (12.2%). The median day for diagnosis of postoperative delirium was postoperative day (POD) 1 (range, 1–3 days). The most common adverse event due to delirium was self-removal of the nasogastric tube by seven patients (Table 2). In one case, accidental self-removal of an ileus tube resulted in intestinal perforation and reoperation. In another case, urethral injury resulted from self-removal of a urethral catheter. One patient removed an epidural anesthesia catheter. Another patient wandered around at night and accidentally fell, resulting in a contusion on his right hip.

**Table 2:** Adverse events due to delirium

Delirium (*n* = 31)	
Adverse events	*n* (%)
Self-removal of nasogastric tube	7 (22.6)
Self-removal of ileus tube	1 (3.2)
Self-removal of urethral catheter	1 (3.2)
Self-removal of epidural anesthesia catheter	1 (3.2)
Fall	1 (3.2)

### Comparison of Peri- Postoperative Factors With and Without Postoperative Delirium

To predict postoperative delirium, we compared the association between postoperative delirium and perioperative factors, including CGA (Table 3). In terms of patient background, the patients with and without postoperative delirium did not differ significantly in terms of age, sex, body mass index (BMI), presence of neoadjuvant chemotherapy, or cStage. The patients with ECOG-PS 2 or 3 were significantly more likely to have delirium (*P* < 0.001). By CGA component, the percentage of patients with frailty was significantly higher based on IADL (*P* = 0.004), MMSE (*P* < 0.001), GDS-15 (*P* < 0.001), and VI (*P* < 0.001) in the group with delirium than in the group without delirium, but there were no significant differences in the Barthel Index (*P* = 0.094).

**Table 3:** Perioperative factors by postoperative delirium status

Characteristic	Postoperative delirium		*P* value
	No	Yes	
	(*n* = 224) *n* (%)	(*n* = 31) *n* (%)	
Age (years)			0.309
Median	79	80	
Range	75–90	75–90	
Sex			0.950
Male	163 (72.8)	23 (74.2)	
Female	61 (27.2)	8 (25.8)	
BMI (kg/m^2^)			0.171
Median	21.9	23.1	
Range	14.3–30.5	16.8–29.6	
ECOG-PS			< 0.001
< 2	211 (94.2)	20 (64.5)	
≥ 2	13 (5.8)	11 (35.5)	
CGA			
Barthel index-frailty	36 (16.1)	9 (29.0)	0.094
IADL–frailty	77 (34.4)	19 (61.3)	0.004
MMSE–frailty	49 (21.9)	23 (74.2)	< 0.001
GDS-15–frailty	47 (21.0)	17 (54.8)	< 0.001
Vitality index-frailty	15 (6.7)	9 (29.0)	< 0.001
Comorbidity			
Hypertension	89 (39.7)	12 (38.7)	0.913
Diabetes mellitus	43 (19.2)	11 (35.5)	0.048
Hyperlipidemia	45 (20.1)	3 (9.7)	0.136
Coronary heart disease	37 (16.5)	10 (32.3)	0.047
Cerebrovascular disorder	28 (12.5)	9 (29.0)	0.025
Respiratory dysfunction	23 (10.3)	4 (12.9)	0.663
Neoadjuvant chemotherapy	14 (6.3)	1 (3.2)	0.470
Clinical stage			0.402
I–II	181 (80.8)	23 (74.2)	
III–IV	43 (19.2)	8 (25.8)	
ASA-PS			0.120
I–II	192 (85.7)	23 (74.2)	
III	32 (14.3)	8 (25.8)	
Surgical procedure			0.455
Open	38 (17.0)	7 (22.6)	
Laparoscopic or robot-assisted	186 (83.0)	24 (77.4)	
Type of gastrectomy			0.444
Distal or proximal	183 (81.7)	27 (87.1)	
Total	41 (18.3)	4 (12.9)	
Operative time (min)			0.364
Median	267	281	
Range	116–608	170–632	
Intraoperative blood loss (ml)			0.059
Median	50	100	
Range	0–1950	5–1550	
Postoperative return destination			0.293
General hospital room	190 (84.8)	24 (77.4)	
Intensive care unit	34 (15.2)	7 (22.6)	
Postoperative hospital stay (days)			< 0.001
Median	16	20	
Range	4–83	8–120	
Discharge status			
Rehabilitation hospital	17 (7.59)	6 (19.4)	0.054
Death	1 (0.5)	2 (6.5)	0.026

BMI, body mass index; ECOG-PS, Eastern Cooperative Oncology Group performance status; CGA, comprehensive geriatric assessment; IADL, Instrumental Activities of Daily Living scale; MMSE, Mini-Mental State Examination; GDS-15, Geriatric Depression Scale 15; ASA-PS, American Society of Anesthesiologists physical status

A higher proportion of the group with delirium had diabetes mellitus (*P* = 0.048), coronary heart disease (*P* = 0.047), and cerebrovascular disorder (*P* = 0.025) than the group without delirium. No significant differences in perioperative patient factors were found for American Society of Anesthesiologists physical status (ASA-PS), surgical procedure, gastrectomy type, operative time, admission to the ICU, or intraoperative blood loss. The postoperative hospital stay was significantly longer for the group with delirium than for the group without delirium (*P* < 0.001). A higher proportion of patients with delirium died during the postoperative hospital stay (*P* = 0.026).

### Multiple Logistic Regression Analysis of Postoperative Delirium: Modeling and Predictors

Factors with a *P* value lower than 0.1 in the univariate analysis were chosen for inclusion in the multivariate analysis. In the multivariate logistic regression analysis, ECOG-PS 2–3 and MMSE-frailty were the only significant independent predictors of postoperative delirium. The odds ratio was 6.59 (95% CI 1.98–21.9; *P* = 0.002) for ECOG-PS 2 or 3 and 7.45 (95% CI 2.81–19.7; *P* < 0.001) for MMSE-frailty (Table 4). When an MMSE score of ≤ 23 and an ECOG-PS score of ≥ 2 were designated as the predictive cutoffs for delirium, sensitivity was 80.7%, and specificity was 74.1% (Table 5).

**Table 4:** Uni- and multivariate analyses of delirium

Variable	Category	Univariate analysis		Multivariate analysis	
		OR (95% CI)	*P* value	OR (95% CI)	*P* value
Age (years)	≥ 80	1.60 (0.75–3.42)	0.227	1.54 (0.59–4.04)	0.379
Sex	Male	1.03 (0.44–2.42)	0.950	1.56 (0.51–4.80)	0.440
ECOG-PS	≥2	8.93 (3.54–22.5)	< 0.001	6.59 (1.98–21.9)	0.002
Barthel index	Frailty	2.14 (0.91–5.02)	0.081	0.52 (0.15–1.90)	0.727
IADL	Frailty	3.02 (1.40–6.55)	0.005	1.21 (0.42–3.51)	0.727
MMSE	Frailty	10.3 (4.33–24.4)	< 0.001	7.45 (2.81–19.7)	< 0.001
GDS-15	Frailty	4.57 (2.10–9.95)	< 0.001	2.50 (0.90–6.95)	0.080
Vitality Index	Frailty	5.70 (2.24–14.5)	< 0.001	2.24 (0.62–8.11)	0.221
Diabetes mellitus	Yes	2.32 (1.03–5.19)	0.042	1.89 (0.68–5.25)	0.225
Coronary heart disease	Yes	2.41 (1.05–5.53)	0.038	1.67 (0.55–5.02)	0.365
Cerebrovascular disorder	Yes	2.86 (1.20–6.84)	0.018	1.13 (0.35–3.70)	0.840
Intraoperative blood loss	>50 ml	2.30 (1.04–5.10)	0.041	2.37 (0.89–6.33)	0.084

OR, odds ratio; CI, confidence interval; ECOG-PS, Eastern Cooperative Oncology Group performance status; IADL, Instrumental Activities of Daily Living scale; MMSE, Mini-Mental State Examination; GDS-15, Geriatric Depression Scale 15

**Table 5:** Diagnostic value of Mini-Mental State Examination (MMSE) score ≤23 or Eastern Cooperative Oncology Group performance status (ECOG-PS) score ≥2 for delirium

MMSE score ≤23 or ECOG-PS ≥2	Delirium		
	(−)	(+)	
(−)	166	6	Sensitivity 80.7% Specificity 74.1% PPV 30.1% NPV 96.5%
(+)	58	25	

PPV, positive predictive value; NPV, negative predictive value

## Discussion

This is the first study to show that CGA and ECOG-PS are predictive of postoperative delirium in elderly patients undergoing gastrectomy for gastric cancer. Delirium was found to be the most common complication after gastrectomy among elderly gastric cancer patients, occurring in 12.2% of patients. The group with delirium was significantly more likely to have ECOG-PS ≥ 2, diabetes mellitus, coronary heart disease, or cerebrovascular disorder. In this group, CGA showed frailty in IADL, MMSE, VI, and GDS-15 items.

In the multivariate analysis, MMSE-frailty and ECOG-PS ≥ 2 were independent risk factors for delirium. Postoperative delirium was more strongly associated with the mental factor of MMSE than with the physical factor of each CGA item or the physical component of ECOG-PS, consistent with our previous reports.^14,15^ The finding that age, operative procedure, and ASA-PS are not predictors of postoperative delirium also is interesting and consistent with previous reports.^14,25^ For the prediction of postoperative delirium, use of an MMSE score of ≤ 23 or an ECOG-PS ≥ 2 as a cutoff resulted in a sensitivity of 80.7% and a specificity of 74.1%. These results suggest that postoperative delirium in elderly patients with gastric cancer might be more easily predicted by a combination of MMSE and ECOG-PS.

Delirium is a clinical diagnosis often unrecognized and overlooked.^9^ The main diagnostic features include acute onset and variable symptom course, inattention, impaired consciousness, and cognitive impairment.^26^ The CAM algorithm is the method most widely used for identification. It is said to have a sensitivity of 94% and specificity of 89%. It is highly reliable.^23^ The CAM algorithm was used for diagnosis in this study.

The primary choice for managing delirium symptoms involves non-pharmacologic approaches such as tapering or discontinuation of psychotropic medications; addressing acute medical issues such as infection, dehydration, abnormal glucose metabolism, and malnutrition; and adjusting circadian rhythms. The use of antipsychotic medications is recommended only for patients with severe agitation that might lead to treatment discontinuation.^9,27^ In this study, a multidisciplinary team consisting of geriatricians, psychiatrists, and other specialists intervened immediately after the diagnosis of postoperative delirium. Of 31 patients, 7 required haloperidol or another antipsychotic medication.

Consistent with previous reports, the group with postoperative delirium in this study had a significantly longer hospital stay (*P* < 0.001).^28^ Therefore, avoiding delirium is not only a medical safety issue, but might also be a way to reduce medical costs due to hospitalization and to avoid ADL declines due to prolonged hospitalization.^29^ In addition, proper preoperative assessment of the risk for delirium might help surgeons explain the risks to patients and families and help families better understand the recovery process and potential outcomes.

In this study, the most common adverse event due to postoperative delirium was self-extraction of tubes implanted in the body. Because the nasogastric tube is important as a drain and source of information about postoperative bleeding, and because intragastric decompression can reduce stress on the anastomosis, self-removal of the nasogastric tube might have a negative impact on postoperative management. Furthermore, in one case of ileus in this study, tube removal resulted in gastrointestinal perforation and reoperation followed by death due to aspiration pneumonia. Such adverse events cannot be ignored in terms of medical safety.

It has been reported that postoperative delirium is preventable in 30–40% of cases.^9^ If patients at high risk for postoperative delirium are identified early, adverse events might be prevented via more comprehensive intervention by a multidisciplinary team that includes psychiatrists, geriatricians, nurses, and rehabilitation specialists.^30^

Regarding the prediction of postoperative delirium, several studies have investigated the usefulness of CGA for assessing the risk of postoperative complications in elderly patients. Yamamoto et al.^14^ reported that MMSE and GDS-15 are important for predicting preoperative delirium in patients with esophageal cancer. In addition to MMSE and GDS-15, Arita et al.^15^ reported that preoperative grip strength measurement is useful for predicting delirium in colorectal cancer. Thus, assessing the risk of delirium in advance and intervening might help reduce delirium. Indeed, it has been reported that in hip arthroplasty, a multidisciplinary preoperative CGA evaluation and intervention by a geriatrician can reduce the risk of postoperative delirium.^31,32^ Moreover, it also has been reported that preoperative administration of antipsychotic drugs reduces the incidence of delirium and has a positive effect on the severity and duration of delirium.^33,34^ Therefore, appropriate identification of patients at high risk for postoperative delirium might allow for proactive therapeutic interventions and prevent delirium from occurring.

Although CGA is important for preoperative risk assessment for the elderly, in reality, not all hospitals have geriatricians on staff, and not all facilities perform CGA evaluation due to the time and cost involved. In fact, the CGA test in this study required approximately 30 min. Due to scheduling conflicts, 93 patients were unable to undergo CGA evaluation. Thus, if implemented as a routine procedure in all hospitals, it could be problematic in terms of time and human resources.

On the other hand, ECOG-PS is a very standard preoperative physical assessment tool, and many hospitals perform it routinely. The MMSE also is more convenient, requiring only approximately 10 min of evaluation by a non-expert geriatrician. Therefore, it is feasible to perform these two tests in a busy daily practice. They might be realistically feasible tools for predicting delirium.

This study had several limitations. First, because this was a retrospective study conducted at a single institution, it had some potential for bias. In the future, a prospective validation study will be conducted with a multidisciplinary team intervention for patients at high risk for postoperative delirium.

Second, several other methods besides CGA can be used to assess surgical risk for elderly patients with cancer, but we have not evaluated or compared them.^35,36^ In the future, it is necessary to compare evaluation tools and search for the best evaluation method.

In conclusion, this study suggests that mental assessment based on the MMSE among CGA items and physical assessment based on ECOG-PS might be important for predicting postoperative delirium in elderly patients with gastric cancer.

## Data Availability

The datasets used and/or analyzed during the current study are available from the corres ponding author on reasonable request.

## References

[1] Artinyan A, Orcutt ST, Anaya DA, Richardson P, Chen GJ, Berger DH. Infectious postoperative complications decrease long-term survival in patients undergoing curative surgery for colorectal cancer: a study of 12,075 patients. *Ann Surg*. 2015;261:497–505. 25185465 10.1097/SLA.0000000000000854

[2] Kataoka K, Takeuchi H, Mizusawa J, et al. Prognostic impact of postoperative morbidity after esophagectomy for esophageal cancer: exploratory analysis of JCOG9907. *Ann Surg*. 2017;265:1152–7. 27280509 10.1097/SLA.0000000000001828

[3] Kurokawa Y, Yamashita K, Kawabata R, et al. Prognostic value of postoperative C-reactive protein elevation versus complication occurrence: a multicenter validation study. *Gastric Cancer*. 2020;23:937–43. 32314097 10.1007/s10120-020-01073-5

[4] Hamel MB, Henderson WG, Khuri SF, Daley J. Surgical outcomes for patients aged 80 and older: morbidity and mortality from major noncardiac surgery. *J Am Geriatr Soc*. 2005;53:424–9. 15743284 10.1111/j.1532-5415.2005.53159.x

[5] Kirkhus L, Šaltytė Benth J, Rostoft S, et al. Geriatric assessment is superior to oncologists’ clinical judgement in identifying frailty. *Br J Cancer*. 2017;117:470–7. 28664916 10.1038/bjc.2017.202 PMC5558687

[6] Rubenstein LZ, Josephson KR, Wieland GD, English PA, Sayre JA, Kane RL. Effectiveness of a geriatric evaluation unit: a randomized clinical trial. *N Engl J Med*. 1984;311:1664–70. 6390207 10.1056/NEJM198412273112604

[7] Yamasaki M, Maekawa Y, Sugimoto K, et al. Development of a geriatric prognostic scoring system for predicting survival after surgery for elderly patients with gastrointestinal cancer. *Ann Surg Oncol*. 2019;26:3644–51. 31388777 10.1245/s10434-019-07687-z

[8] Yamashita K, Yamasaki M, Makino T, et al. Preoperative comprehensive geriatric assessment predicts postoperative risk in older patients with esophageal cancer. *Ann Surg Oncol*. 2023;30:901–9. 36367627 10.1245/s10434-022-12778-5

[9] Inouye SK, Westendorp RGJ, Saczynski JS. Delirium in elderly people. *Lancet*. 2014;383:911–22. 23992774 10.1016/S0140-6736(13)60688-1 PMC4120864

[10] Francis J, Martin D, Kapoor WN. A prospective study of delirium in hospitalized elderly. *JAMA*. 1990;263:1097–101. 2299782

[11] Saczynski JS, Marcantonio ER, Quach L, et al. Cognitive trajectories after postoperative delirium. *N Engl J Med*. 2012;367:30–9. 22762316 10.1056/NEJMoa1112923 PMC3433229

[12] Yanagimoto Y, Kurokawa Y, Doki Y. Essential updates 2021/2022: perioperative and surgical treatments for gastric and esophagogastric junction cancer. *Ann Gastroenterol Surg*. 2023;7:698–708. 37663969 10.1002/ags3.12711 PMC10472390

[13] Kurokawa Y, Kawase T, Takeno A, et al. Phase 2 trial of neoadjuvant docetaxel, oxaliplatin, and S-1 for clinical stage III gastric or esophagogastric junction adenocarcinoma. *Ann Gastroenterol Surg*. 2023;7:247–54. 36998295 10.1002/ags3.12632 PMC10043771

[14] Yamamoto M, Yamasaki M, Sugimoto K, et al. Risk evaluation of postoperative delirium using comprehensive geriatric assessment in elderly patients with esophageal cancer. *World J Surg*. 2016;40:2705–12. 27272271 10.1007/s00268-016-3602-2

[15] Arita A, Takahashi H, Ogino T, et al. Grip strength as a predictor of postoperative delirium in patients with colorectal cancers. *Ann Gastroenterol Surg*. 2022;6:265–72. 35261952 10.1002/ags3.12519 PMC8889853

[16] Maekawa Y, Sugimoto K, Yamasaki M, et al. Comprehensive geriatric assessment is a useful predictive tool for postoperative delirium after gastrointestinal surgery in old-old adults. *Geriatr Gerontol Int*. 2016;16:1036–42. 26311242 10.1111/ggi.12587

[17] Mahoney FI, Barthel DW. Functional evaluation: the Barthel Index. *Md State Med J*. 1965;14:61–5. 14258950

[18] Lawton MP, Brody EM. Assessment of older people: self-maintaining and instrumental activities of daily living. *Gerontologist*. 1969;9:179–86. 5349366

[19] Holsinger T, Deveau J, Boustani M, Williams JW Jr. Does this patient have dementia? *JAMA*. 2007;297:2391–404. 17551132 10.1001/jama.297.21.2391

[20] Yesavage JA, Brink TL, Rose TL, et al. Development and validation of a geriatric depression screening scale: a preliminary report. *J Psychiatr Res*. 1982;17:37–49. 7183759 10.1016/0022-3956(82)90033-4

[21] Azam F, Latif MF, Farooq A, et al. Performance status assessment by using ECOG (Eastern Cooperative Oncology Group) score for cancer patients by oncology healthcare professionals. *Case Rep Oncol*. 2019;12:728–36. 31616281 10.1159/000503095 PMC6792426

[22] Japanese Gastric Cancer Association. Japanese Gastric Cancer Treatment Guidelines 2021 (6th edition). *Gastric Cancer*. 2023;26:1–25. 10.1007/s10120-022-01331-8 PMC9813208 36342574

[23] Wong CL, Holroyd-Leduc J, Simel DL, Straus SE. Does this patient have delirium? *Value of bedside instruments JAMA*. 2010;304:779–86. 20716741 10.1001/jama.2010.1182

[24] Dindo D, Demartines N, Clavien PA. Classification of surgical complications: a new proposal with evaluation in a cohort of 6336 patients and results of a survey. *Ann Surg*. 2004;240:205–13. 15273542 10.1097/01.sla.0000133083.54934.ae PMC1360123

[25] Kristjansson SR, Nesbakken A, Jordhøy MS, et al. Comprehensive geriatric assessment can predict complications in elderly patients after elective surgery for colorectal cancer: a prospective observational cohort study. *Crit Rev Oncol Hematol*. 2010;76:208–17. 20005123 10.1016/j.critrevonc.2009.11.002

[26] Inouye SK, van Dyck CH, Alessi CA, Balkin S, Siegal AP, Horwitz RI. Clarifying confusion: the confusion assessment method: a new method for detection of delirium. *Ann Intern Med*. 1990;113:941–8. 2240918 10.7326/0003-4819-113-12-941

[27] Marcantonio ER. Postoperative delirium: a 76-year-old woman with delirium following surgery. *JAMA*. 2012;308:73–81. 22669559 10.1001/jama.2012.6857 PMC3604975

[28] Gleason LJ, Schmitt EM, Kosar CM, et al. Effect of delirium and other major complications on outcomes after elective surgery in older adults. *JAMA Surg*. 2015;150:1134–40. 26352694 10.1001/jamasurg.2015.2606 PMC4684425

[29] O’Mahony R, Murthy L, Akunne A, Young J, Guideline Development Group. Synopsis of the National Institute for Health and Clinical Excellence guideline for prevention of delirium. *Ann Intern Med*. 2011;154:746–51. 10.7326/0003-4819-154-11-201106070-00006 21646557

[30] Janssen TL, Steyerberg EW, Langenberg JCM, et al. Multimodal prehabilitation to reduce the incidence of delirium and other adverse events in elderly patients undergoing elective major abdominal surgery: an uncontrolled before-and-after study. *PLoS One*. 2019;14:e0218152. 31194798 10.1371/journal.pone.0218152 PMC6564537

[31] Stenvall M, Berggren M, Lundström M, Gustafson Y, Olofsson B. A multidisciplinary intervention program improved the outcome after hip fracture for people with dementia: subgroup analyses of a randomized controlled trial. *Arch Gerontol Geriatr*. 2012;54(:e284–9. 10.1016/j.archger.2011.08.013 21930310

[32] Deschodt M, Braes T, Flamaing J, et al. Preventing delirium in older adults with recent hip fracture through multidisciplinary geriatric consultation. *J Am Geriatr Soc*. 2012;60:733–9. 22429099 10.1111/j.1532-5415.2012.03899.x

[33] Larsen KA, Kelly SE, Stern TA, et al. Administration of olanzapine to prevent postoperative delirium in elderly joint-replacement patients: a randomized, controlled trial. *Psychosomatics*. 2010;51:409–18. 20833940 10.1176/appi.psy.51.5.409

[34] Schrader SLP, Wellik KE, Demaerschalk BM, Caselli RJ, Woodruff BK, Wingerchuk DM. Adjunctive haloperidol prophylaxis reduces postoperative delirium severity and duration in at-risk elderly patients. *Neurologist*. 2008;14:134–7. 18332845 10.1097/NRL.0b013e318166b88c

[35] Bellera CA, Rainfray M, Mathoulin-Pélissier S, et al. Screening older cancer patients: first evaluation of the G-8 geriatric screening tool. *Ann Oncol*. 2012;23:2166–72. 22250183 10.1093/annonc/mdr587

[36] Huisman MG, Ghignone F, Ugolini G, et al. Long-term survival and risk of institutionalization in onco-geriatric surgical patients: long-term results of the PREOP study. *J Am Geriatr Soc*. 2020;68:1235–41. 32155289 10.1111/jgs.16384 PMC7318670

